# Rules for the Exhibition of the Letheon

**Published:** 1847-10

**Authors:** 


We had intended selecting from the various amount of matter written on the use of the Ether some two articles for this No. of the Register, but the article from Dr. J. Taylor, Maysville, answers our object fully as well, for it presents both views of the subject, and is the result of a very extended practice. Our use of the article has been rather limited, and we still prefer operating without it. Yet we may say that the cases in which we have used it, it has prove succsssful.

We append the following in relation to its exhibition from "Wood's Quarterly Retrospect":

RULES FOR EXHIBITION.

1st. The ether employed should be the purest washed sulphuric ether.

2d. The patient should be allowed to respire atmospheric air alone for a few moments, if the apparatus is so formed as to allow of it; if not, the nose should not be closed, until several respirations have been taken, and the patient begins to breathe without trepidation.

3d. The ether should not be turned on in a full jet at once, but the stopcock should be so regulated as gradually to accustom the bronchial tubes to the vapor.

At this time coughing is apt to ensue, especially if the ether be not perfectly pure; this symptom, however, soon subsides, or can be moderated by regulating the jet of the ether.

4th. Surgeons differ in opinion as to the exact point at which inhalation should be suspended; we believe that for surgical purposes, Mr. Robinson's test, as afforded by the state of the eye, will be a sufficiently good guide.

5th. In prolonged operations, it is necessary to alternate respiration of pure atmospheric air, with that of ether vapor; this is accomplished by removing the clip from the nose, or, still better, in those instruments which are so made, by turning off the ether and turning on the air.—*Ranking's Abstract, July,* 1847.

TIME REQUIRED TO PRODUCE INSENSIBILITY.

This varies mainly, we believe, according to the degree of skill with which the vapor is exhibited. We have seen it produced in two minutes, and only imperfectly induced at the expiration of twenty; in the latter instances we have generally observed some imperfection in the instrument, or in the application of the mouth piece. Insensibility is more rapidly produced in children and women than in men, and the period appears to be abridged by repetition of the inhalation.

PERIOD DURING WHICH INSENSIBILITY REMAINS.

This is also subject to variation, the average duration may be stated to be from two to six minutes. Sometimes, and especially in those ill-managed cases in which the patient is more suffocated than etherized, he does not perfectly recover his consciousness for half an hour or more. The restoration is sometimes gradual, at others sudden, the patient instantly starting up as from a dream. He is for a moment or two, somewhat incoherent, and staggers about as if half drunk. No ill effects are left behind in the majority of cases; but in some, more or less headache remains for the rest of the day.—*Ranking's Abstract, July,* 1847.

